# Supplementary material for: Nature experiences affect the aesthetic reception of art: The case of paintings depicting aquatic animals
Source: PLoS One. 2024 Jul 18;19(7):e0303584. doi: 10.1371/journal.pone.0303584 (PMC11257337; doi:10.1371/journal.pone.0303584)

S1: **Semantic analysis of the representations of the paintings according to the observers.**
It is based on written statements of what each painting evoked to observers (‘*In a few words, what does this painting evoke for you?*’)
Each statement has been divided into semantic units, classified according to relevant categories (e.g., ‘richness of the sea’, ‘wonders’, abundance’, and ‘diversity’ have been classified into the same category).
The diagrams represent the percentage of appearance of semantic units in each category (e.g., P1 has been interpreted as a ‘still-life’ in 29% of the statements).

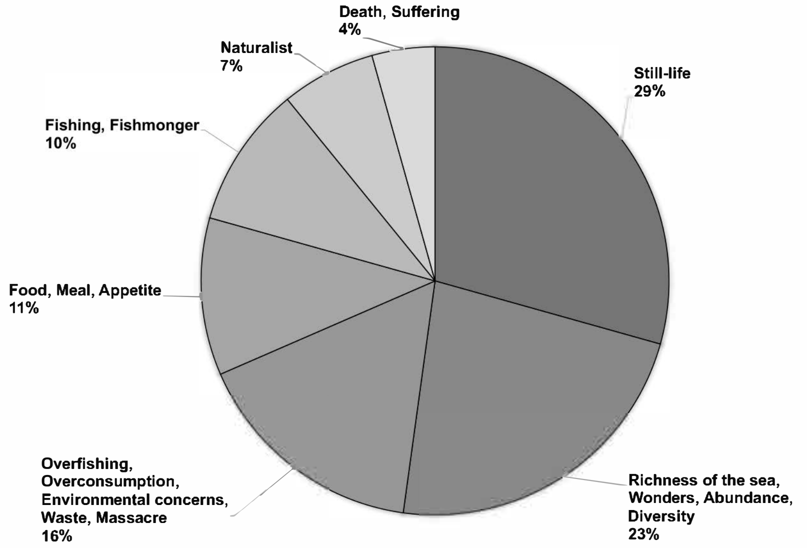

P1: Giuseppe Recco - Naples (Italy) 1634 - Alicante (Spain) 1695, *Pisces*, 1683. Private collection.

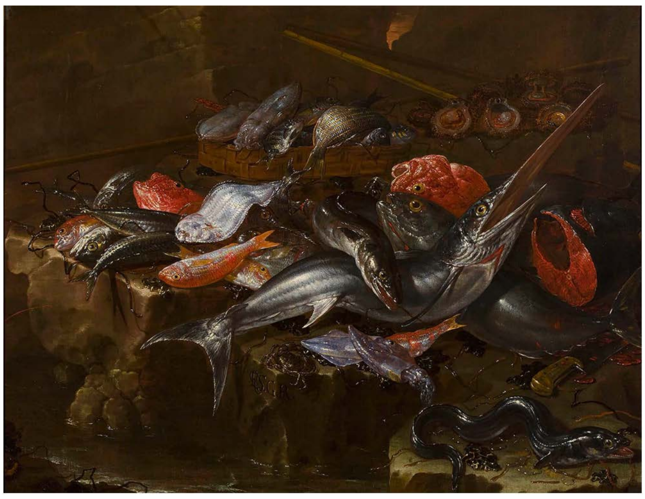

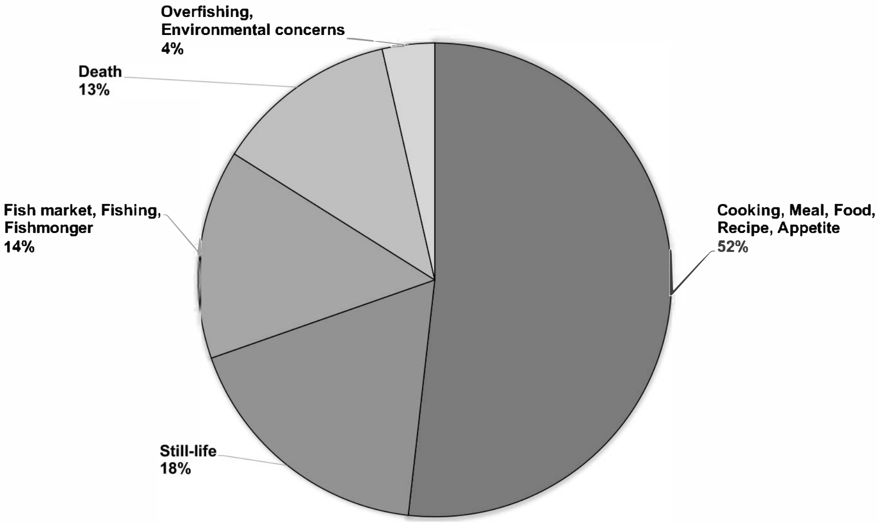

P2: Antonio Viladomat - Barcelona (Spain) 1678 - Barcelona (Spain) 1755, *Still Life with Shellfish, Fish and Vessels*, 1710-1740. Prado National Museum. Source: ©Photographic Archive Museo Nacional del Prado.

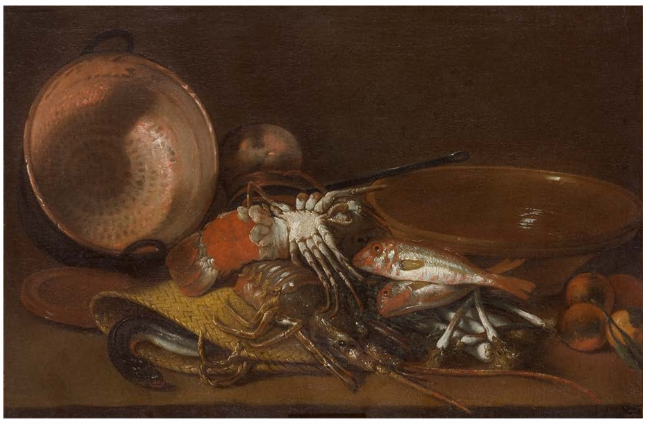

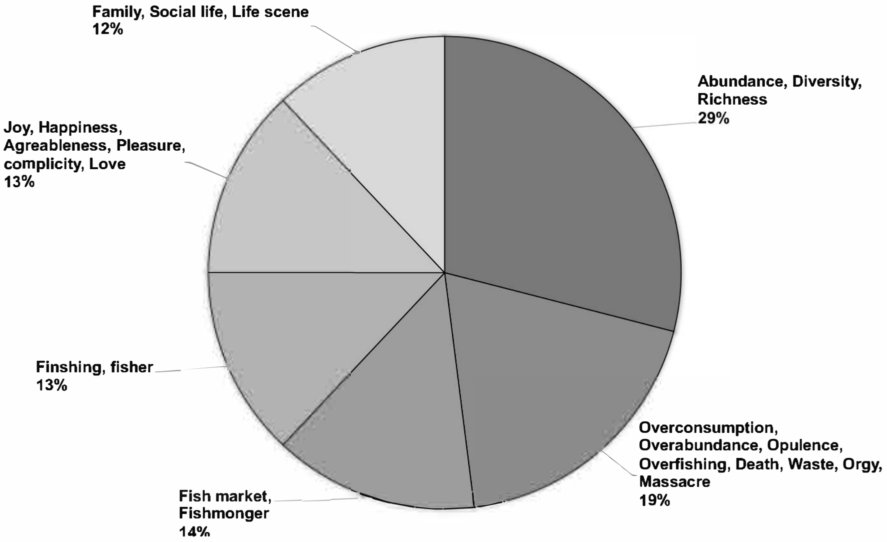

P3: Vincenzo Campi - Cremona (Italy) 1536 - Cremona (Italy) 1591, *The fishmongers*, 1579. Museum of La Roche-sur-Yon. Public domain CC0.

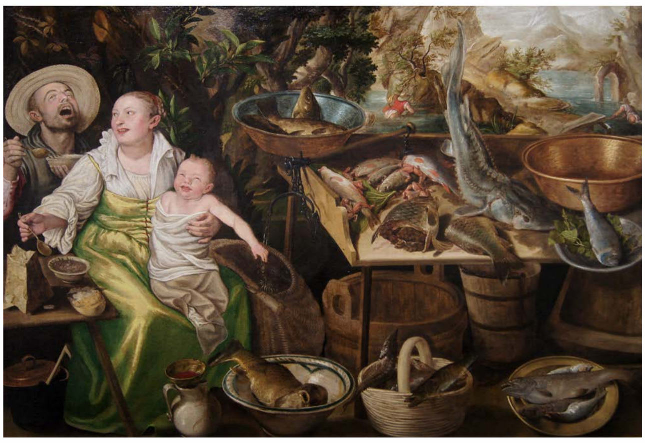

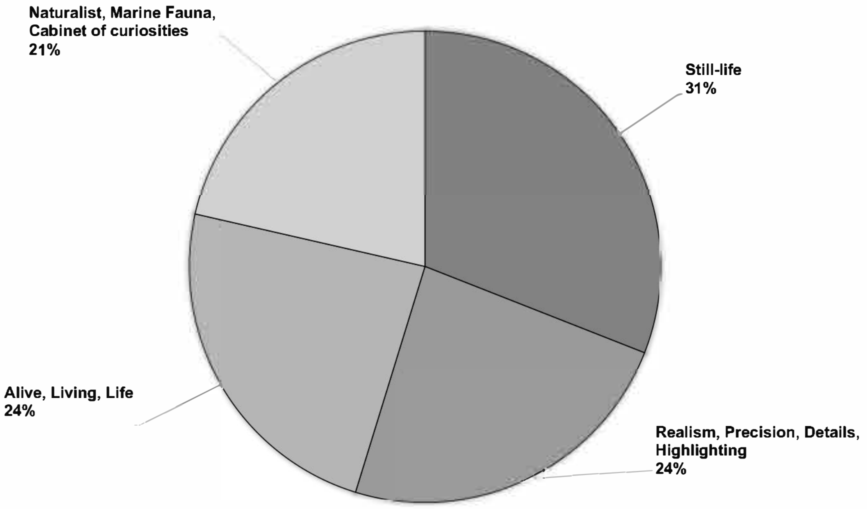

P4: Paolo Porpora - Naples (Italy) 1617 - Rome (Italy) 1673, Still life of fish and crustaceans. Public domain CC0.

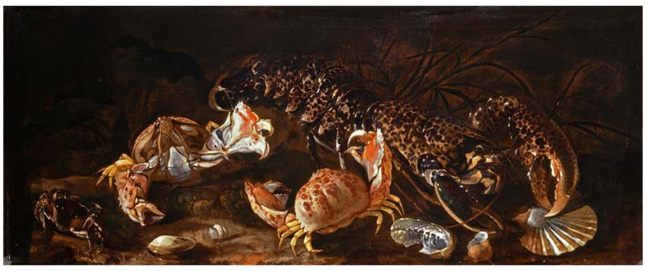

Supplement: S1 File — (PDF) [file pone.0303584.s001.pdf]
