# Supplementary material for: Nature experiences affect the aesthetic reception of art: The case of paintings depicting aquatic animals
Source: PLoS One. 2024 Jul 18;19(7):e0303584. doi: 10.1371/journal.pone.0303584 (PMC11257337; doi:10.1371/journal.pone.0303584)
Supplement: S2 File — (PDF) [file pone.0303584.s002.pdf]

S2: Questionnaire. Translated from French to English.

Aesthetic reception score (ARS)

To what extent do you agree with the following statements? (strongly agree, somewhat agree, neither agree nor disagree, somewhat disagree, completely disagree) :

This painting is pleasant
This painting disgusts me
This painting makes me feel afraid
This painting is beautiful
This painting makes me feel troubled
This painting features a high level of creativity
The artists manner of painting is fascinating
The composition of this painting is of high quality
This painting is very innovative
This painting is unique
It is exciting to think about this painting
This painting makes me sad
This painting makes me feel lonesome
This painting makes me feel angry
I can associate this painting with my own personal biography
This painting makes me think about my own life history
Personal memories of mine are linked to this painting
This painting makes me curious
This painting is thought-provoking
It is fun to deal with this painting
I can relate this painting to a particular artist
I know this painting
I can relate this painting to its art historical context
In a few words, what does this painting evoke for you?

Fish Comsumption Scores (EAT) items

How frequently do you eat fish? daily–several times a week–weekly
several times a month–monthly–less than monthly–never

To what extent do you agree with the following statements? (strongly agree, somewhat agree, neither agree nor disagree, somewhat disagree, completely disagree) :

I have much knowledge about fish
I find it difficult to judge the quality of fish (reversescaled)
I am familiar with preparing fish.
I am very satisfied when fish is on the menu
Fish has a good taste
Fish has an unpleasant smell (reverse-scaled)
The bones in fish are unpleasant (reverse-scaled)
Eating fish is healthy
Eating fish is safe
Fish is difficult to prepare (reverse-scaled)
Eating fish is expensive (reverse-scaled)
Fish is easily available for me

Marine experiences

How many years have you lived in a coastal area or by the sea?
Do you go fishing or spearfishing at sea? (yes, no)
Do you practice scuba-diving or snorkelling at sea? (yes, no)

Aesthetic sensitivity

Over the past twelve months, how many times have you visited a temporary exhibition or a museum? (none, once or twice, three or four, five or more)
Do you have a job or a professional project related to the visual arts? (yes, no)

Additional Information

How old are you ?
What is your gender ?
What is your socio-professional category?
Do you have a job or professional project related to the environment? (yes, no)
