# Supplementary material for: Nature experiences affect the aesthetic reception of art: The case of paintings depicting aquatic animals
Source: PLoS One. 2024 Jul 18;19(7):e0303584. doi: 10.1371/journal.pone.0303584 (PMC11257337; doi:10.1371/journal.pone.0303584)
Supplement: S3 File — (PDF) [file pone.0303584.s003.pdf]

S3: Exploratory Factor Analysis of Aesthetic reception scale and Fish consumption scale.

Exploratory Factor Analyses (oblimin rotation)

Aesthetic Reception Score (ARS) items	Factor contribtion						Item Parameters				
	1	2	3	4	5	6	Uniqueness	α	Item-rest correlation	Mean	Sd
This painting is pleasant	-.758						.364	.796	.079	1.885	1.129
This painting disgusts me	.716						.380	.801	.021	1.106	1.264
This painting makes me feel afraid	.641						.454	.796	.100	1.308	1.210
This painting is beautiful	-.622						.477	.786	.265	2.417	1.131
This painting makes me feel troubled	.597						.503	.788	.243	1.503	1.246
This painting features a high level of creativity		.748					.435	.774	.495	2.046	1.039
The artists manner of painting is fascinating		.612					.491	.772	.524	2.288	1.082
The composition of this painting is of high quality		.604					.541	.777	.459	2.752	.922
This painting is very innovative		.561					.714	.783	.351	2.028	.726
This painting is unique		.561					.486	.769	.544	2.057	1.263
It is exciting to think about this painting		.483					.378	.770	.547	2.015	1.167
This painting makes me sad			.927				.128	.790	.195	.966	1.169
This painting makes me feel lonesome			.712				.494	.792	.134	.630	.962
This painting makes me feel angry			.686				.430	.786	.252	.652	1.032
I can associate this painting with my own personal biography				.824			.336	.777	.422	.888	1.193
This painting makes me think about my own life history				.817			.332	.777	.427	.791	1.108
Personal memories of mine are linked to this painting				.755			.404	.776	.431	1.080	1.312
This painting makes me curious					.805		.252	.770	.531	2.413	1.221
This painting is thought-provoking					.616		.487	.768	.565	2.037	1.261
It is fun to deal with this painting					.421		.504	.781	.359	2.230	1.284
I can relate this painting to a particular artist						.759	.410	.784	.302	.637	.989
I know this painting						.551	.658	.787	.245	.472	1.016
I can relate this painting to its art historical context						.530	.685	.788	.252	1.759	1.301

Fish Comsumption Scores (EAT) items	Factor contribtion				Item Parameters				
	1	2	3	4	Uniqueness	α	Item-rest correlation	Mean	Sd
I have much knowledge about fish	.864				.312	.748	.475	1.991	1.287
I find it difficult to judge the quality of fish (reversescaled)	.835				.304	.764	.324	2.426	1.111
I am familiar with preparing fish.	.575				.294	.722	.692	2.092	1.329
I am very satisfied when fish is on the menu		.882			.241	.748	.480	2.666	1.190
Fish has a good taste		.613			.609	.755	.433	3.301	.915
How frequently do you eat fish? *daily–several times a week–weekly several times a month–monthly–less than monthly–never*		.473			.572	.747	.488	3.052	1.556
Fish has an unpleasant smell (reverse-scaled)		.360			.803	.760	.370	2.178	1.227
The bones in fish are unpleasant (reverse-scaled)					.854	.768	.281	1.132	1.138
Eating fish is healthy			.693		.472	.769	.247	2.911	.856
Eating fish is safe			.655		.560	.770	.263	1.825	1.153
Fish is difficult to prepare (reverse-scaled)				.547	.627	.764	.324	2.426	1.111
Eating fish is expensive (reverse-scaled)				.506	.756	.780	.114	1.301	.970
Fish is easily available for me					.789	.760	.364	2.650	1.181
