# Supplementary material for: Nature experiences affect the aesthetic reception of art: The case of paintings depicting aquatic animals
Source: PLoS One. 2024 Jul 18;19(7):e0303584. doi: 10.1371/journal.pone.0303584 (PMC11257337; doi:10.1371/journal.pone.0303584)
Supplement: S4 File — (PDF) [file pone.0303584.s004.pdf]

S4: Confirmatory Factor Analysis of Aesthetic reception scale and Fish consumption scale.

Confirmatory Factor Analyses

ARS Factors	Items	Estimate	SE	Z	p	Model parameters	
ATTRACTIVITY	This painting is beautiful	.855	.0415	2.6	< .001	CFI	.866
	This painting is pleasant	.973	.0406	24.0	< .001	TLI	.843
ARTISTIC QUALITY	The composition of this painting is of high quality	.601	.0345	17.4	< .001	RMSE	.0782
	This painting is very innovative	.327	.0291	11.2	< .001		
	This painting features a high level of creativity	.751	.0376	2.0	< .001		
	The artists manner of painting is fascinating	.792	.0389	2.3	< .001		
	This painting is unique	.917	.0457	2.1	< .001		
NEGATIVE EMOTIONALITY	This painting makes me feel afraid	.921	.0430	21.4	< .001		
	This painting makes me feel troubled	.841	.0462	18.2	< .001		
	This painting disgusts me	.980	.0447	21.9	< .001		
	This painting makes me sad	.833	.0434	19.2	< .001		
	This painting makes me feel lonesome	.532	.0385	13.8	< .001		
	This painting makes me feel angry	.740	.0381	19.4	< .001		
EXPERTISE	I know this painting	.592	.0472	12.5	< .001		
	I can relate this painting to a particular artist	.742	.0491	15.1	< .001		
	I can relate this painting to its art historical context	.697	.0591	11.8	< .001		
SELF-REFERENCE	I can associate this painting with my own personal biography	.963	.0421	22.9	< .001		
	Personal memories of mine are linked to this painting	1.024	.0468	21.9	< .001		
	This painting makes me think about my own life history	.895	.0391	22.9	< .001		
COGNITIVE STIMULATION	It is exciting to think about this painting	.956	.0400	23.9	< .001		
	It is fun to deal with this painting	.901	.0468	19.3	< .001		
	This painting is thought-provoking	.617	.0509	12.1	< .001		
	This painting makes me curious	.916	.0439	2.9	< .001		

EAT Factors	Items	Estimate	SE	Z	p	Model parameters	
PAST EXPERIENCE	I have much knowledge about fish	0.984	0.0478	20.58	< .001	CFI	.981
	I find it difficult to judge the quality of fish (reversescaled)	0.993	0.0459	21.64	< .001	TLI	.956
	I am familiar with preparing fish	1.090	0.0503	21.69	< .001	RMSE	.0543
AFFECTIVE JUDGEMENTS	I am very satisfied when fish is on the menu	0.998	0.0481	20.73	< .001		
	Fish has a good taste	0.564	0.0369	15.29	< .001		
	Fish has an unpleasant smell (reverse-scaled)	0.501	0.0532	9.42	< .001		
	The bones in fish are unpleasant (reverse-scaled)	0.280	0.0503	5.57	< .001		
EVALUATIVE JUDGEMENTS	Eating fish is healthy	0.699	0.0627	11.15	< .001		
	Eating fish is safe	0.675	0.0682	9.89	< .001		
PERCEIVED BEHAVIORAL CONTROL	Fish is difficult to prepare (reverse-scaled)	0.517	0.0620	8.34	< .001		
	Eating fish is expensive (reverse-scaled)	0.213	0.0549	3.88	< .001		
	Fish is easily available for me	0.656	0.0696	9.42	< .001		
BEHAVIOR	How frequently do you eat fish? *daily–several times a week–weekly several times a month–monthly–less than monthly–never*	1.556	0.0432	36.03	< .001		
