# Supplementary material for: Nature experiences affect the aesthetic reception of art: The case of paintings depicting aquatic animals
Source: PLoS One. 2024 Jul 18;19(7):e0303584. doi: 10.1371/journal.pone.0303584 (PMC11257337; doi:10.1371/journal.pone.0303584)
Supplement: S5 File — (PDF) [file pone.0303584.s005.pdf]

S5: Principal Component Analysis of Marine activities, Artistic sensitivity and Sociodemographic variables.

Principal Compenent Analysis (varimax rotation)

**Factors**	**Variable**	**Component contributions** 1	2	3	4	**Uniqueness**
CP1: Age + SPC	Socioprofessional category (SPC)	.812				.287
	Age (Age)	.720		-.324		.315
CP2: FSH	Fishing (Fsh)		.780			.331
	Littoral (Litt)	.346	.716			.352
CP3: DIV	Professional activity related to environment (P.env)			.834		.297
	Diving (Div)		.314	.672		.442
CP4: ART	Museum visit (Mus)				.767	.404
	Professional activity related to visual arts (P.art)				.757	.419
	% variance	17.0	16.3	16.2	14.9	
	% variance (cumulative)	17.0	33.3	49.5	64.4	
