# Supplementary material for: Nature experiences affect the aesthetic reception of art: The case of paintings depicting aquatic animals
Source: PLoS One. 2024 Jul 18;19(7):e0303584. doi: 10.1371/journal.pone.0303584 (PMC11257337; doi:10.1371/journal.pone.0303584)
Supplement: Attachment — Submitted filename: 4-Responses after rejection for editorial reasons.doc [file pone.0303584.s006.doc]

Dr Thomas Changeux

Mediterranean Institute of Oceanography (MIO)

Campus de Luminy case 901 163 Av. de Luminy 13288 Marseille cedex 9

FRANCE

[thomas.changeux@ird.fr](mailto:thomas.changeux@ird.fr)

GSM :+33 6 59 80 87 48

PLOS One

1265 Battery Street, Suite 200
San Francisco, CA 94111
United States

Subject: PLOS ONE Decision: PONE-D-23-28152R1 - [EMID:6507259d54a45917]

Dear Dr Karmakar,

Last December you have rejected our manuscript in reference because you were feeling that it didn’t comply with the ethical requirements for human subject research submissions of PLOS One.

After an in-depth examination of the issue you have raised, we suggest that you reconsider your position in the light of the new information below completed in response to the summary of requirements of your journal <http://journals.plos.org/plosone/s/human-subjects-research>)..

1-Obtain prior approval for human subjects research by an institutional review board (IRB) or equivalent ethics committee(s)

>>We can now provide you with the CNIL (French national commission for data processing and freedoms) declaration of conformity to the reference methodology framework MR-001, received on December 5, 2023, knowing that the ethic committee of Aix-Marseille University has adopted the following (translation from <https://www.univ-amu.fr/fr/public/comite-dethique>).

“2-OUTSIDE THE JARDE LAW AND THE ETHICS COMMITTEE'S JURISDICTION

Research involving the human person within the meaning of II of article R1121-1 of the Public Health Code (...) - opinion surveys, pure observations in the social, ethnological or ethological sciences.”

Our on line questionnaire study is clearly an opinion survey and do not enter ethic committee jurisdiction.

2-Submit documentation from the review board or ethics committee confirming approval of the research. Identifying information about study participants must be redacted from the approval document before it is submitted to the journal”

>> We do not have any information enabling to identify the participants, as the online questionnaire was anonymous.

3-Declare compliance with ethical practices upon submission of a manuscript

>>You will find in the end of this letter a copy of PLOS One Human Participants Research Checklist we have signed and kept at your disposal.

4-Report details on how informed consent for the research was obtained (or explain why consent was not obtained)

>> Participants gave their informed consent by checking a box at the beginning of the questionnaire stating: “Knowing the information transmitted to me, I freely and voluntarily agree to participate in the research project entitled: “Aesthetic reception of aquatic biodiversity in art.””

5-For clinical trials, provide trial registration details, the study protocol, and CONSORT documentation (more information below)

>> It is not a clinical trial.

6-Confirm that an identified individual has provided written consent for the use of that information

>> We confirm that, even if they are not identifiable, the participants have given their consent by ticking the box as indicated in previous §4.

Our manuscript had already been the subject of a previous review (PONE-D-23-28152R1, to which we responded to each point raised by the reviewers), but following your comments, we have modified it slightly (line 205-207).

If you agree to reconsider your decision, we offer you the opportunity to submit a new version and the original .pdf documents mentioned in this letter.

Sincerely,

Thomas CHANGEUX, Ph D

----------------------------------------------------------------------------------------------------------------------------------------

Copy of PLOS One One Human Participants Research Checklist

Complete the following if your study involved human participants or human participants’ data. These questions should be addressed for prospective and retrospective studies.

1.Did you obtain ethics approval for this study?

•If yes, please upload (file type “Other”) the original approval document you received from your ethics committee. If the original document is in another language, please also provide an English translation.

>>English translation of the document:

“CNIL (national commission for data processing and freedoms) declaration of conformity

Declaration of conformity to the reference methodology framework MR-001, received on December 5, 2023

By this declaration, the declarant certifies the conformity of his/her processing(s) of personal data with the reference system mentioned above. The CNIL may at any time verify, by mail or by means of an on-site or online inspection, the conformity of this processing(s).”

X Uploaded

2. If you prospectively recruited human participants for the study – for example, you conducted a clinical trial, distributed questionnaires, or obtained tissues, data or samples for the purposes of this study, please report in the Methods:

i. the day, month and year of the start and end of the recruitment period for this study.

>> The study was carried out from September 2022 to November 2022 (see L. 205)

ii.whether participants provided informed consent, and if so, what type was obtained (for instance, written or verbal, and if verbal, how it was documented and witnessed). If your study included minors, state whether you obtained consent from parents or guardians. If the need for consent was waived by the ethics committee, please include this information.

>> The information is now included L. 205 – 207 ou our new manuscript: “The study was carried out on an adult audience (over 18 years old), recruited by an email information campaign among Aix-Marseille University staff and national academic mailing lists. Participants gave their informed consent by checking a box at the beginning of the questionnaire stating: Knowing the information transmitted to me, I freely and voluntarily agree to participate in the research project entitled: “Aesthetic reception of aquatic biodiversity in art”.”

_X__ Completed	___ N/A

3.If you are reporting a retrospective study of medical records or archived samples, please report in the Methods section:

i. the day, month and year when the data were accessed for research purposes

ii. whether authors had access to information that could identify individual participants during or after data collection

___ Completed	_X__ N/A: not concerned

Signed Thomas CHANGEUX
